# Supplementary material for: Bioactivity Profiles of Cytoprotective Short-Chain Quinones
Source: Molecules. 2021 Mar 4;26(5):1382. doi: 10.3390/molecules26051382 (PMC7961879; doi:10.3390/molecules26051382)
Supplement: Supplementary file 1 [file molecules-26-01382-s001.pdf]

Supplementary Materials

Bioactivity Profiles of Cytoprotective Short-Chain Quinones

Zikai Feng [1,2,†], Monila Nadikudi [1,†], Krystel L. Woolley [2], Ayman L. Hemasa [1], Sueanne Chear [1], Jason A. Smith [2], and Nuri Gueven [1,*]

1 School of Pharmacy and Pharmacology, University of Tasmania, Hobart, TAS 7005, Australia; zikai.feng@utas.edu.au (Z.F.); monila.nadikudi@utas.edu.au (M.N.); ayman.hemasa@utas.edu.au (A.L.H.); sueanne.chear@utas.edu.au (S.C.)

2 School of Natural Sciences, University of Tasmania, Hobart, TAS 7005, Australia; krystel.woolley@utas.edu.au (K.L.W.); jason.smith@utas.edu.au (J.A.S.)

* Correspondence: nuri.guven@utas.edu.au

† Authors contributed equally to this manuscript.

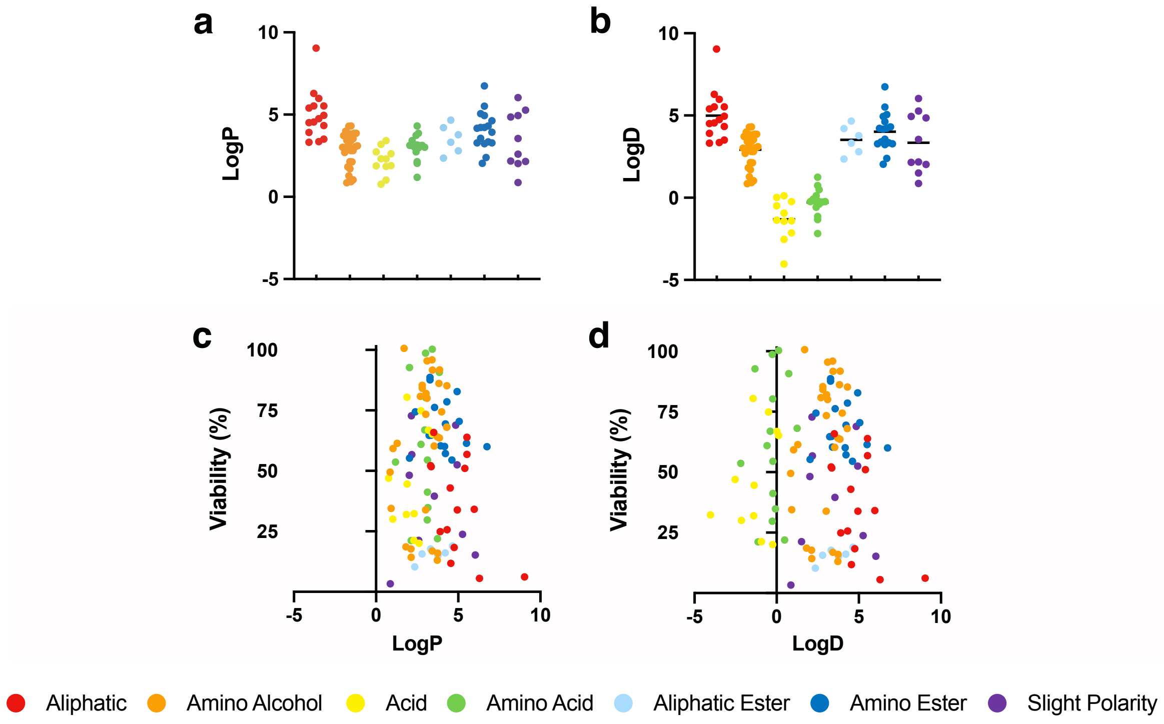

Figure S1. Physical properties of short-chain quinone (SCQ) test compounds. (**a**) Partition coefficient (logP), (**b**) distribution coefficient (logD) and (**c**-**d**) their correlations with SCQ-protected HepG2 cell viability. Partition coefficient (logP) of test compounds was predicted using ChemDraw Professional software (version 16.0, PerkinElmer, Waltham, MA, USA). Distribution coefficient (logD) of test compounds was predicted using MarvinView software (version 19.25, ChemAxon, Budapest, Hungary).

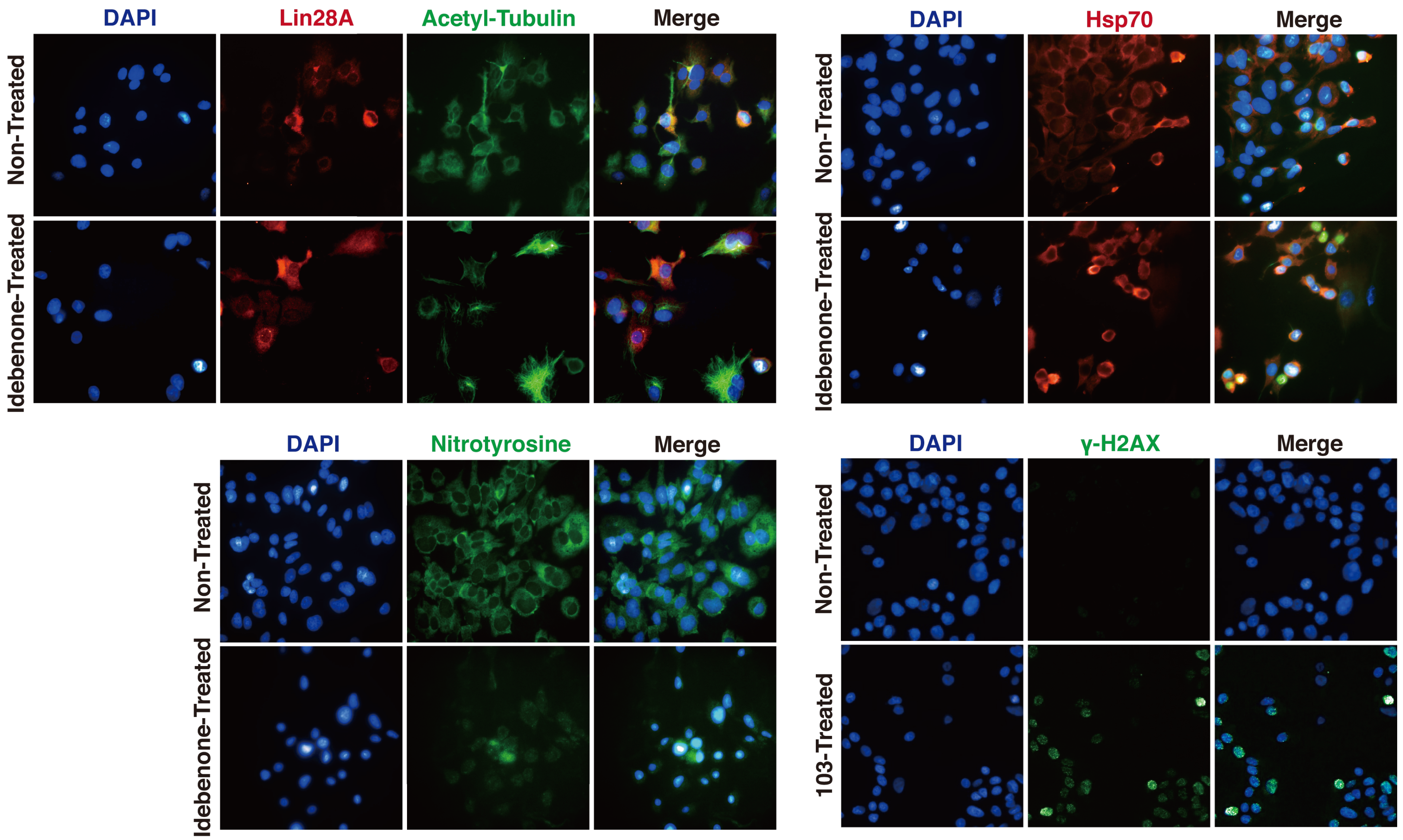

Figure S2. Exemplary images of stained HepG2 cells. Methods detailed in the main text sections 4.6-4.8 and 4.10-4.11. Images acquired using an IN Cell 2200 analyzer (60 × magnification, GE Healthcare, Rydalmere, NSW, Australia).

Table S1. Synthesis of novel SCQs.

SCQ	Synthesis	^{1}H NMR δ (CDCl$_3$, 400 MHz)	^{13}C NMR δ (CDCl$_3$, 100 MHz)	[α]$_D^{20}$	IR V$_{max}$
1	**1** was prepared according to General Procedure B (see caption for details) from **4** (100 mg, 0.4240 mmol) and tyramine (66 mg, 0.4818 mmol) and the product purified by flash chromatography (90% ethyl acetate/hexane) to give **1** as yellow viscous oil in 51% yield (77 mg, 0.2158 mmol).	1.68 (quin, *J* = 7.4 Hz, 2H), 1.97 (s, 9H), 2.18 (t, *J* = 7.4 Hz, 2H), 2.41–2.45 (m, 2H), 2.72 (t, *J* = 7.0 Hz, 2H), 3.45–3.50 (m, 2H), 6.08 (t, *J* = 5.7 Hz, 1H), 6.75 (d, *J* = 8.3 Hz, 2H), 6.97 (d, *J* = 8.4 Hz, 2H), 7.54 (bs, 1H)	12.2, 12.42, 12.48, 24.5, 25.9, 34.7, 36.2, 41.0, 115.6, 129.7, 129.9, 140.4, 140.8, 141.1, 143.3, 155.3, 173.1, 187.5, 187.7	-	3362 (N-H), 2937, 1641 (C=O), 1516, 1456, 1374, 1262, 832, 716 cm^{-1}
2	**2** was prepared according to General Procedure B from **4** (96 mg, 0.4053 mmol) and 3,4-dimethoxyphenethylamine (107 mg, 0.5926 mmol) and the product purified by flash chromatography (90% ethyl acetate/hexane) to give **2** as yellow semi solid in 42% yield (69 mg, 0.1717 mmol).	1.67 (quin, *J* = 7.7 Hz, 2H), 1.95 (s, 3H), 1.96 (s, 3H), 1.99 (s, 3H), 2.15 (t, *J* = 7.2 Hz, 2H), 2.41–2.45 (m, 2H), 2.73 (t, *J* = 7.0 Hz, 2H), 3.45–3.50 (m, 2H), 3.80 (s, 3H), 3.81 (s, 3H), 5.84 (t, *J* = 5.1 Hz, 1H), 6.68–6.70 (m, 2H), 6.75 (d, 8.6 Hz, 1H)	12.2, 12.3, 12.4, 24.4, 25.9, 35.5, 36.1, 40.7, 55.8, 55.9, 111.4, 111.9, 120.6, 131.4, 140.3, 140.6, 140.9, 143.3, 147.7, 149.0, 172.3, 187.3, 187.6	-	3375 (N-H), 3300, 2936, 1642 (C=O), 1455, 1374, 1261, 1236, 1157, 1140, 1028, 846, 717 cm^{-1}
3	**3** was prepared according to General Procedure B from **4** (182 mg, 0.7692 mmol) and L-phenylalaninol (193 mg, 1.277 mmol) and the product purified by flash chromatography (2% methanol/ethyl acetate) to give **4** as yellow viscous oil in 11% yield (52 mg, 0.1352 mmol).	1.63–1.71 (m, 2H), 2.01 (s, 9H), 2.20 (t, *J* = 7.1 Hz, 2H), 2.24 (t, *J* = 7.8 Hz, 2H), 2.89 (t, *J* = 7.0 Hz, 2H), 3.59 (dd, *J* = 11.0, 5.2 Hz, 1H), 3.71 (dd, *J* = 11.0, 3.5 Hz, 1H), 4.19–4.27 (m, 1H), 6.14 (d, *J* = 7.8 Hz, 1H), 7.18–7.29 (m, 5H)	12.2, 12.41, 12.49, 24.4, 25.8, 36.2, 37.1, 52.9, 64.3, 126.6, 128.6, 129.2, 137.8, 140.4, 140.9, 141.1, 143.3, 173.0, 187.62, 187.63	-26.91° (*c* 0.54, CHCl$_3$)	3374 (N-H), 3299, 2936, 1642 (C=O), 1538, 1455, 1374, 1042, 845, 702 cm^{-1}
4	**4** was prepared according to General Procedure A (see caption for details) from trimethyl-*p*-benzoquinone (411 mg, 2.738 mmol) and glutaric acid (728 mg, 5.512 mmol) and the product purified by flash chromatography (100% CH_2Cl_2 followed by 100% ethyl acetate) to give **4** as a crystalline yellow solid in 33% yield (216 mg, 0.9136 mmol) with a melting point of 56–57°C.	1.69 (quin, *J* = 7.4 Hz, 2H), 1.95 (s, 6H), 1.98 (s, 3H), 2.36 (t, *J* = 7.4 Hz, 2H), 2.47–2.51 (m, 2H)	12.1, 12.35, 12.38, 23.5, 25.8, 33.7, 140.5, 140.6, 140.9, 143.1, 179.2, 187.0, 187.6	-	2940, 1707 (C=O), 1642 (C=O), 1457, 1375, 1260, 1158, 717 cm^{-1}
5	**5** was prepared from the deprotection of **6** (87 mg, 0.1976 mmol) using General Procedure C (see caption for details). The product was purified by flash chromatography (5% methanol/ethyl acetate) to give **5** as brown solid in a quantitative yield with a melting point of 72–74°C.	1.54–1.60 (m, 2H), 1.89 (s, 3H), 1.90 (s, 3H), 1.91 (s, 3H), 2.16 (t, *J* = 7.1 Hz, 2H), 2.31–2.34 (m, 2H), 3.03 (dd, *J* = 14.0, 7.2 Hz, 1H), 3.18 (dd, *J* = 14.0, 5.1 Hz, 1H), 4.77 4.81 (m, 1H), 6.51 (d, *J* = 7.3 Hz, 1H), 7.08–7.09 (m, 2H), 7.13–7.20 (m, 3H)	12.2, 12.4, 12.5, 24.3, 25.7, 35.7, 37.2, 53.6, 127.4, 128.8, 129.3, 135.7, 140.5, 141.0, 141.4, 143.0, 174.2, 174.6, 187.6, 187.7	+32.78° (*c* 1.88, CHCl$_3$)	3355 (N-H), 2934, 1738 (C=O), 1717 (C=O), 1642 (C=O), 1539, 1456, 1375, 1206, 1172, 702 cm^{-1}
6	**6** was prepared according to General Procedure B from **4** (253 mg, 1.072 mmol) and L-phenylalanine *t*-butyl ester·HCl (284 mg, 1.105 mmol) and the product purified by flash chromatography (40% ethyl acetate/hexane) to give **6** as yellow viscous oil in 24% yield (111 mg, 0.2531 mmol).	1.41 (s, 9H), 1.71 (quin, *J* = 7.4 Hz, 2H), 2.01 (s, 6H), 2.02 (s, 3H), 2.22 (t, *J* = 7.4 Hz, 2H), 2.48 (t, *J* = 8.4 Hz, 2H), 3.09–3.11 (m, 2H), 4.77 (q, *J* = 6.7 Hz, 1H), 6.07 (d, *J* = 7.6 Hz, 1H), 7.15–7.29 (m, 5H)	12.2, 12.3, 12.4, 24.3, 25.9, 28.0, 36.0, 38.1, 53.5, 82.3, 126.9, 128.4, 129.5, 136.3, 140.4, 140.6, 140.9, 143.4, 170.9, 171.7, 187.2, 187.7	+47.24° (*c* 0.58, CHCl$_3$)	3303 (N-H), 2978, 2933, 1738 (C=O), 1717 (C=O), 1645 (C=O), 1538, 1456, 1368, 1155, 701 cm^{-1}
7	**7** was prepared according to General Procedure B from **10** (85 mg, 0.3161 mmol) and tyramine (48 mg, 0.3477 mmol) and the product purified by flash chromatography (2% methanol/ethyl acetate) to give **7** as yellow viscous oil in 13% yield (16 mg, 0.0413 mmol).	1.70 (quin, *J* = 7.4 Hz, 2H), 2.01 (s, 3H), 2.17 (t, *J* = 7.4 Hz, 2H), 2.43–2.47 (m, 2H), 2.75 (t, *J* = 7.0 Hz, 2H), 3.47–3.52 (m, 2H), 3.987 (s, 3H), 3.988 (s, 3H), 5.26 (bs, 1H), 5.62 (d, *J* = 4.9 Hz, 1H), 6.76 (d, *J* = 8.5 Hz, 2H), 7.04 (d, *J* = 8.5 Hz, 2H)	12.1, 24.4, 25.7, 34.7, 35.9, 41.1, 61.3 (2 × C), 115.6, 129.9, 130.4, 139.9, 141.8, 144.4, 144.6, 154.8, 172.9, 184.58, 184.5	-	3348 (N-H), 3281, 2944, 1652 (C=O), 1645 (C=O), 1611, 1516, 1456, 1265, 1204, 1053, 844, 737 cm^{-1}

**8**	**8** was prepared according to General Procedure B from **10** (96 mg, 0.3590 mmol) and 3,4-dimethoxyphenethylamine (107 mg, 0.5926 mmol) and the product purified by flash chromatography (90% ethyl acetate/hexane) to give **8** as yellow viscous oil in 16% yield (25 mg, 0.0586 mmol).	1.71 (quin, *J* = 7.4 Hz, 2H), 2.02 (s, 3H), 2.17 (t, *J* = 7.4 Hz, 2H), 2.44–2.68 (m, 2H), 2.77 (t, *J* = 7.0 Hz, 2H), 3.49–3.53 (m, 2H), 3.85 (s, 3H), 3.86 (s, 3H), 3.98 (s, 6H), 5.61 (t, *J* = 5.1 Hz, 1H), 6.72–6.74 (m, 2H), 6.79–6.81 (m, 1H)	12.1, 24.4, 25.7, 35.3, 36.1, 40.8, 56.01, 56.05, 61.2, 61.3, 111.5, 112.0, 120.7, 131.4, 139.7, 141.9, 144.4, 144.5, 147.8, 149.2, 172.2, 184.4, 184.5	-	3371 (N-H), 3302, 2938, 1652 (C=O), 1645 (C=O), 1645, 1516, 1455, 1263, 1237, 1156, 1028, 846 cm^{-1}
**9**	**9** was prepared according to General Procedure B from **10** (77 mg, 0.2818 mmol) and L-phenylalaninol (102 mg, 0.6779 mmol) and the product purified by flash chromatography (2% methanol/ethyl acetate) to give **9** as yellow oil in 17% yield (19 mg, 0.0483 mmol).	1.68 (quin, *J* = 7.4 Hz, 2H), 1.99 (s, 3H), 2.17 (t, *J* = 7.4 Hz, 2H), 2.39–2.43 (m, 2H), 2.87 (t, *J* = 6.5 Hz, 2H), 3.58 (dd, *J* = 11.0, 5.1 Hz, 1H), 3.71 (dd, *J* = 11.0, 3.5 Hz, 1H), 3.97 (s, 3H), 3.98 (s, 3H), 4.18–4.22 (m, 1H), 5.91 (d, *J* = 7.5 Hz, 1H), 7.18–7.21 (m, 3H), 7.27–7.29 (m, 2H)	12.0, 24.3, 25.5, 36.0, 37.1, 53.0, 61.3 (2 × C), 64.3, 126.7, 128.7, 129.3, 137.8, 139.8, 141.9, 144.4, 144.6, 172.8, 184.55, 184.56	-42.76° (*c* 0.29, $CHCl_3$)	3356 (N-H), 3293, 2942, 1645 (C=O), 1610, 1456, 1265, 1205, 1045, 702 cm^{-1}
**10**	**10** was prepared according to General Procedure A from 2,3-dimethoxy-5-methyl-*p*-benzoquinone (478 mg, 2.622 mmol) and glutaric acid (756 mg, 5.724 mmol) and the product purified by flash chromatography (30% ethyl acetate/hexanes followed by 100% ethyl acetate) to give **10** as a yellow oil in 25% yield (177 mg, 0.6598 mmol).	1.70 (quin, *J* = 7.7 Hz, 2H), 1.99 (s, 3H), 2.37 (t, *J* = 7.1 Hz, 2H), 2.47–2.51 (m, 2H), 3.94 (s, 6H)	11.9, 23.4, 25.6, 33.5, 61.21, 61.22, 139.6, 141.7, 144.41, 144.47, 178.5, 184.0, 184.5	-	3340 (-OH), 2950, 1706 (C=O), 1649 (C=O), 1611, 1456, 1266, 1206, 1154, 1105, 1059, 745 cm^{-1}
**11**	**11** was prepared from the deprotection of **12** (11 mg, 0.0231 mmol), using General Procedure C. The product was purified by flash chromatography (5% methanol/ethyl acetate) to give **11** as yellow viscous oil in 84% yield (8 mg, 0.0195 mmol).	1.59 (quin, *J* = 7.7 Hz, 2H), 1.93 (s, 3H), 2.13–2.25 (m, 2H), 2.28–2.44 (m, 2H), 2.92 (dd, *J* = 14.0, 9.4 Hz, 1H), 3.22 (dd, *J* = 14.0, 5.0 Hz, 1H), 3.952 (s, 3H), 3.955 (s, 3H), 4.67 (dd, *J* = 9.4, 5.0 Hz, 1H), 7.15–7.27 (m, 5H)	12.2, 25.9, 26.8, 36.6, 38.7, 55.3, 61.8, 61.9, 128.0, 129.7, 130.5, 138.9, 140.9, 143.4, 146.1, 146.2, 175.1, 175.5, 185.7, 186.2	+57.27° (*c* 0.22, $CHCl_3$)	3350 (N-H), 2947, 1733 (C=O), 1652 (C=O), 1645 (C=O), 1611, 1456, 1266, 1204, 1153, 1055, 735, 702 cm^{-1}
**12**	**12** was prepared according to General Procedure B from **10** (101 mg, 0.3761 mmol) and L-phenylalanine t-butyl ester·HCl (108 mg, 0.4190 mmol) and the product purified by flash chromatography (40% ethyl acetate/hexane) to give **12** as yellow viscous oil in 12% yield (21 mg, 0.0437 mmol).	1.40 (s, 9H), 1.70 (quin, *J* = 7.5 Hz, 2H), 2.00 (s, 3H), 2.21 (t, *J* = 7.5 Hz, 2H), 2.43–2.47 (m, 2H), 3.08 (d, *J* = 6.0 Hz, 2H), 3.98 (s, 6H), 4.72–4.77 (m, 1H), 5.96 (d, *J* = 7.6 Hz, 1H), 7.13–7.28 (m, 5H)	12.0, 24.3, 25.7, 28.0, 36.0, 38.2, 53.5, 61.2 (2 × C), 82.5, 127.0, 128.5, 129.5, 136.3, 139.6, 142.0, 144.50, 144.57, 170.9, 171.7, 184.2, 184.6	+40.78° (*c* 0.25, $CHCl_3$)	3369 (N-H), 2978, 2940, 1729 (C=O), 1652 (C=O), 1647, 1611, 1456, 1368, 1266, 1205, 1153, 1055, 701 cm^{-1}
**13**	**13** was prepared according to General Procedure A from 1,4-naphthoquinone (0.530 g, 3.351 mmol) and 4-methyl valeric acid (1.476 g, 12.71 mmol). Purification by flash column chromatography (50% CH_2Cl_2/hexanes) resulted in **13** being identified as a yellow crystalline solid in 35% yield (271 mg, 1.188 mmol) with a melting point of 35–38°C.	0.95 (d, *J* = 6.7 Hz, 6H), 1.42–1.48 (m, 2H), 1.65 (septet, *J* = 6.7 Hz, 1H), 2.57 (td, *J* = 8.1, 1.4 Hz, 2H), 6.78 (t, *J* = 1.4 Hz, 1H), 7.70–7.73 (m, 2H), 8.04–8.06 (m, 1H), 8.08–8.10 (m, 1H)	22.5, 27.6, 28.1, 37.2, 126.1, 126.7, 132.2, 132.5, 133.72, 133.75, 134.7, 152.4, 185.3, 185.7	-	2956, 2928, 2870, 1662 (C=O), 1620, 1595, 1467, 1367, 1329, 1301, 1265, 779 cm^{-1}
**14**	**14** was prepared according to General Procedure B from **17** (150 mg, 0.6149 mmol) and tyramine (89 mg, 0.6502 mmol) and the product purified by flash chromatography (80% ethyl acetate/hexanes) to give **14** as a brown viscous oil in 33% yield (109 mg, 0.2885 mmol).	1.85 (quin, *J* = 7.4 Hz, 2H), 2.22 (t, *J* = 7.4 Hz, 2H) 2.49–2.53 (m, 2H), 2.72 (t, *J* = 6.8 Hz, 2H), 3.46–3.50 (m, 2H), 5.89 (t, *J* = 5.27 Hz, 1H), 6.71 (s, 1H), 6.77 (d, *J* = 8.4 Hz, 2H), 6.99 (d, *J* = 8.4 Hz, 2H), 7.68–7.71 (m, 2H), 7.99–8.05 (m, 2H)	24.0, 29.0, 34.7, 35.9, 40.9, 115.7, 121.7, 126.1, 126.7, 129.9, 132.1, 132.2, 133.8, 133.9, 150.9, 155.1, 172.6, 185.1, 185.24, 185.28	-	3312 (N-H), 2932, 1661 (C=O), 1594, 1539, 1455, 1302, 1265, 1042, 733, 702 cm^{-1}
**15**	**15** was prepared according to General Procedure B from **17** (140 mg, 0.5748 mmol) and 3,4-dimethoxyphenethylamine (129 mg, 0.7111 mmol) and the product purified by flash chromatography (90% ethyl acetate/hexanes) to give **15** as brown semi solid in 36% yield (84 mg, 0.2059 mmol).	1.90 (quin, *J* = 7.4 Hz, 2H), 2.21 (t, *J* = 7.4 Hz, 2H), 2.54–2.58 (m, 2H), 7.26 (t, *J* = 7.1 Hz, 2H), 3.47–3.52 (m, 2H), 3.83 (s, 3H), 3.85 (s, 3H), 5.63 (t, *J* = 5.5 Hz, 2H), 6.71 (s, 1H), 6.77–6.80 (m, 3H), 7.76–7.74 (m, 2H), 8.03–8.08 (m, 2H)	24.0, 29.1, 35.3, 35.9, 40.7, 56.00, 56.03, 111.5, 112.0, 120.7, 126.2, 126.7, 131.4, 132.2, 132.3, 133.7, 133.8, 135.2, 147.8, 149.2, 150.9, 172.0, 185.0, 185.2	-	3311 (n-H), 2935, 1644 (C=O), 1593, 1516, 1328, 1302, 1263, 1236, 1157, 1141, 1028, 732 cm^{-1}

**16**	**16** was prepared according to General Procedure B from **17** (140 mg, 0.5728 mmol) and L-phenylalaninol (96 mg, 0.6349 mmol) and the product purified by flash chromatography (4% methanol/ethyl acetate) to give **16** as a dark brown semi solid in 53% yield (115 mg, 0.3034 mmol).	1.86 (quin, *J* = 7.3 Hz, 2H), 2.25 (t, *J* = 7.3 Hz, 2H), 2.49–2.53 (m, 2H), 2.89 (t, *J* = 7.0 Hz, 2H), 3,04 (bs, 1H), 3.59 (dd, *J* = 11.1, 5.1 Hz, 1H), 3.70 (dd, *J* = 11.1, 3.6 Hz, 1H), 4.21–4.25 (m, 1H), 6.17 (d, *J* = 7.9 Hz, 1H), 6.76 (s, 1H), 7.19–7.29 (m, 5H), 7.71–7.73 (m, 2H), 8.02–8.07 (m, 2H)	24.9, 28.9, 35.9, 37.1, 52.8, 64.0, 126.1, 126.7, 128.6 (2 × C), 129.3 (2 × C), 132.1, 132.2, 133.8, 135.3, 137.8, 150.9, 172.7, 185.1, 185.3	-23.09° (*c* 0.97, $CHCl_3$)	3350 (N-H), 2947, 1733 (C=O), 1645 (C=O), 1611, 1456, 1266, 1204, 1153, 1055, 735, 702 cm^{-1}
**17**	**17** was prepared according to General Procedure A from 1,4-naphthoquinone (1.999 g, 12.64 mmol) and glutaric acid (0.8354 mg, 6.323 mmol) and the product purified by a Reveleris® X2 automated flash chromatography system (eluent: gradient 0–80% ethyl acetate in hexane; column: Reveleris® Silica 24 g; flow rate: 18 mL/min) to give **17** as a brown solid in 42% yield (0.655 g, 2.680 mmol) with a melting point of 120–122°C.	1.90 (quin, *J* = 7.6 Hz, 2H), 2.39 (t, *J* = 7.6 Hz, 2H), 2.62 (td, *J* = 7.6, 1.1 Hz, 2H), 6.85 (t, *J* = 1.1 Hz, 1H), 7.78–7.80 (m, 2H), 8.02–8.04 (m, 1H), 8.07–8.10 (m, 1H)	24.4, 30.0, 34.2, 126.8, 127.4, 133.4, 133.7, 134.8, 134.9, 136.0, 152.4, 176.8, 186.1, 186.3	-	2956 (-OH), 1699 (C=O), 1660 (C=O), 1620, 1953, 1417, 1327, 1303, 1265, 1143, 783, 661 cm^{-1}
**18**	A solution of gamma amino butyric acid (170 mg, 1.6466 mmol) in H2O (5 mL) was added to a hot solution of 1,4-naphthoquinone (525 mg, 3.317 mmol) in ethanol (50 mL) and the mixture stirred at room temperature for 16 h. The solvent removed under reduced pressure and the product purified by a Reveleris® X2 automated flash chromatography system (eluent: gradient 0–10% methanol in CH_2Cl_2; column: Reveleris® Silica 4 g; flow rate: 18 mL/min) to give **18** as a burgundy red solid in 22% yield (92 mg, 0.3560 mmol).	2.04 (quin, *J* = 7.0 Hz, 2H), 2.50 (t, *J* = 7.0 Hz, 2H), 3.29 (q, *J* = 7.0 Hz, 2H), 5.78 (s, 1H), 6.07–6.10 (m, 1H), 7.61 (td, *J* = 7.5, 1.3 Hz, 1H), 7.72 (td, *J* = 7.5, 1.3 Hz, 1H), 8.03 (dd, *J* = 7.7, 1.0 Hz, 1H), 8.09 (dd, *J* = 7.7, 1.0 Hz, 1H)	22.6, 30.9, 41.22, 99.3, 125.2, 125.8, 130.4, 132.0, 133.1, 134.7, 148.5, 174.1, 181.2, 181.5	-	-
**19**	**19** was prepared from the deprotection of **20** (428 mg, 0.9563 mmol), using General Procedure C. The product was purified by flash chromatography (5% methanol/ethyl acetate) to give **19** as brown solid in 92% yield (345 mg, 0.8811 mmol) with a melting point of 110–112°C.	1.86 (quin, *J* = 7.4 Hz, 2H), 2.40 (t, *J* = 7.3 Hz, 2H), 2.45–2.54 (m, 2H), 3.12 (dd, *J* = 14.0, 7.4 Hz, 1H), 3.28 (dd, *J* = 14.0, 5.2 Hz, 1H), 4.97–5.02 (m, 1H), 6.74 (s, 1H), 7.04 (d, *J* = 7.9 Hz, 1H), 7.17–7.21 (m, 3H), 7.24–7.28 (m, 2H), 7.71–7.73 (m, 2H), 7.99–8.06 (m, 2H)	24.0, 28.8, 35.3, 37.3, 53.5, 126.2, 126.7, 127.4, 128.8, 129.3, 131.9, 132.1, 134.0, 135.3, 135.53, 135.55, 150.7, 174.82, 174.88, 185.2, 185.6	+7.87° (*c* 0.33, MeOH)	3301 (N-H), 2929, 1708 (C=O), 1661 (C=O), 1554, 1454, 1369, 1302, 1260, 697 cm^{-1}
**20**	**20** was prepared according to General Procedure B from **17** (351 mg, 1.437 mmol) and L-phenylalanine t-butyl ester·HCl (496 mg, 1.925 mmol) and the product purified by flash chromatography (50% ethyl acetate/hexanes) to give **20** as yellow viscous oil in 67% yield (432 mg, 0.9660 mmol).	1.43 (s, 9H), 1.91 (quin, *J* = 7.5 Hz, 2H), 2.29 (td, *J* = 7.5, 2.9 Hz, 2H), 2.56–2.60 (m, 2H), 3.10–3.14 (m, 2H), 4.76–4.81 (m, 1H), 6.03 (d, *J* = 7.3 Hz, 1H), 7.16 -6 7.31 (m, 5H), 7.74–7.76 (m, 2H), 8.07–8.12 (m, 2H)	23.9, 28.1, 29.1, 35.8, 38.2, 53.5, 82.5, 126.2, 126.7, 127.1, 128.5 (2 × C), 129.6 (2 × C), 132.2, 132.3, 133.7, 133.8, 135.3, 136.3, 150.9, 170.9, 172.5, 185.1, 185.2	+38.46° (*c* 0.39, $CHCl_3$)	3309 (N-H), 2978, 2931, 1732 (C=O), 1662 (C=O), 1595, 1525, 1456, 1367, 1301, 1259, 1153, 700 cm^{-1}
**21**	**21** was prepared according to General Procedure B from **18** (63 mg, 0.2416 mmol) and L-phenylalanine methyl ester·HCl (60 mg, 0.2791 mmol) in DMF. The reaction was quenched with H_2O (20mL) and the aqueous layer was extracted with 1:1 ethyl acetate/hexanes. The organic layer was combined and washed with 2 × 25 mL H_2O, dried with $MgSO_4$, filtered and the solvent removed under reduced pressure to give a crude product. The product was purified by flash chromatography (90% ethyl acetate/hexanes) to give **21** as bright red solid in 18% yield (19 mg, 0.0445 mmol) with a melting point of 105–107°C.	1.97–2.04 (m, 2H), 2.29–2.33 (m, 2H), 3.08 (dd, *J* = 14.0, 6.2 Hz, 1H), 3.18 (dd, *J* = 14.0, 5.8 Hz, 1H), 3.19–3.23 (m, 2H), 3.73 (s, 3H), 4.90–4.95 (m, 1H), 5.82 (s, 1H), 5.96 (d, *J* = 7.6 Hz, 1H), 6.51 (bs, 1H), 7.07–7.09 (m, 2H), 7.22–7.29 (m, 3H), 7.61 (td, *J* = 7.6, 1.3 Hz, 1H), 7.72 (td, *J* = 7.6, 1.3 Hz), 8.05 (dd, *J* = 7.7, 1.0 Hz, 1H), 8.09 (dd, *J* = 7.7, 1.0 Hz, 1H)	23.4, 33.6, 37.9, 42.4, 52.5, 53.2, 100.5, 126.4, 126.6, 127.3, 128.7 (2 × C), 129.3 (2 × C), 130.6, 132.2, 133.5, 134.9, 135.8, 148.6, 171.6, 172.1,181.6, 182.8	-46.67° (*c* 0.06, $CHCl_3$)	3290 (N-H), 3061, 2953, 1743 (C=O), 1676 (C=O), 1604, 1570, 1510, 1456, 1336, 1305, 1253, 1213, 779 cm^{-1}

**33**	Glutaric acid (4.368 g, 0.0331 mol) was added to a solution of menadione (2.984g, 0.0173 mol) in CH_3CN/H_2O (3:1, 50 mL) and the mixture was heated to 75°C. To this solution, AgNO3 (321 mg, 1.891 mmol) was added followed by the slow addition of $(NH_4)_2S_2O_8$ (9.897 g, 0.0434 mol) in H_2O (20 mL) over 1.5 h. The resulting mixture was stirred for a further 2 h, before being left o/n at room temperature. The mixture was extracted with CH_2Cl_2 (3 × 50 mL) and the organic extract washed with H_2O (4 × 50 mL). The organic layer was dried over $MgSO_4$, filtered and the solvent removed under reduced pressure to give the crude product, which was purified by a Reveleris® X2 automated flash chromatography system (eluent: gradient 100% Hexanes - 100% ethyl acetate; column: Reveleris® Silica 40 g; flow rate: 30 mL/min) to **33** identified as a yellow needle crystals in <1% yield (10 mg, 0.0260 mmol).	1.66–1.74 (m, 2H), 2.20 (s, 6H), 2.76 (t, *J* = 8.0 Hz, 4H), 7.66–7.70 (m, 2H), 8.02–8.07 (m, 2H)	12.8, 27.3, 27.4, 126.3, 126.4, 132.22, 132.26, 133.53, 133.54, 143.7, 146.6, 184.7, 185.2	-	1658 (C=O), 1618, 1595, 1377, 1325, 1296, 711 cm^{-1}
**101**	Following preparation for **13,** product purification by flash column chromatography (50% CH_2Cl_2/hexanes) resulted in **101** being identified as a yellow viscous in a 10% yield (10 mg, 0.0322 mmol).	1.00 (d, *J* = 6.7 Hz, 12H), 1.36–1.41 (m, 4H), 1.72 (ap. Octet, *J* = 6.6 Hz, 2H), 2.59–2.64 (m, 4H), 7.69–7.71 (m, 2H), 8.07–8.09 (m, 2H)	22.5, 25.1, 28.9, 38.7, 126.2, 132.4, 133.3, 147.5, 185.2	-	2956, 2924, 2852, 1660 (C=O), 1597, 1465, 1367, 1317, 1284, 1259, 1103, 719 cm^{-1}
**102**	Following preparation for **17**, product purification by a Reveleris® X2 automated flash chromatography system (eluent: gradient 0–80% ethyl acetate in hexane; column: Reveleris® Silica 24 g; flow rate: 18 mL/min) resulted in **102** as a crystalline yellow solid in 9% yield (185 mg, 0.5607 mmol) with a melting point of 146–149°C.	1.80 (quin, *J* = 7.4 Hz, 4H), 2.42 (t, *J* = 7.4 Hz, 4H), 2.69–2.73 (m, 4H), 7.74–7.76 (m, 2H), 8.03–8.05 (m, 2H)	25.7, 27.2, 34.7, 127.1, 133.5, 134.6, 148.0, 176.9, 186.1	-	2932 (-OH), 1697 (C=O), 1656 (C=O), 1595, 1406, 1294, 1240, 935, 721 cm^{-1}
**103**	**103** was isolated as a by-product from the silver decarboxylation reaction of trifluoroacetic acid with 1,4-nathpthoquinone. The spectral and mass spectrometry data for the compound are consistent with that reported by Betts *et al*. HRMS (EI) calculated 174.0317 (M+), found 174.0313.	4.01 (s, 3H), 7.78 (m, 2H), 8.10 (m, 2H)	($CDCl_3$, 75 MHz) 55.40, 127.31, 131.86, 134.81, 190.82	-	16951 (C=O), 1595, 1325, 1294, 858, 719 cm^{-1}

General Procedure A (silver-mediated radical decarboxylation general method): carboxylic acid (2 equiv.) was added to a solution of menadione (1 equiv.) in CH_3CN/H2O (3:1) and the mixture was heated to 75 °C. To this solution, $AgNO_3$ (0.1 equiv.) was added followed by the slow addition of $(NH_4)_2S_2O_8$ (2.5 equiv.) in H_2O (5 mL) over 10 min. The resulting mixture was stirred for a further 1 h. The mixture was cooled to room temperature (RT), extracted with CH_2Cl_2 and the organic extract washed with saturated $NaHCO_3$ and H_2O. The organic layer was dried over $MgSO_4$, filtered and the solvent removed under reduced pressure to give the crude product, which was purified by flash chromatography (silica gel). **General Procedure B** (quinone amide coupling general method): quinone acid (1 equiv.) was added to anhydrous dichloromethane (5–10 mL) under an atmosphere of N_2 and cooled to 0°C. Amino acid (1 equiv.), dimethyl aminopyridine (DMAP, 0.1 equiv.), triethylamine (Et_3N, 2.5 equiv.) and either EDCI, BOP or PyBOP (1.4 equiv.) were added successively and the reaction mixture warmed slowly to RT before leaving overnight. The reaction was quenched with H_2O (20 mL) and the organic layer washed with saturated $KHSO_4$ solution, saturated $NaHCO_3$ solution and H_2O. The organic layer was dried with $MgSO_4$, filtered and the solvent removed under reduced pressure to give a crude product, which was purified by flash chromatography (silica gel) to give the amide. **General Procedure C** (*t*-butyl ester deprotection method): the *t*-butyl esters were added to 10% TFA in CH_2Cl_2 (5.0 mL) and the reaction mixture stirred at RT overnight and the solvent removed under reduced pressure. The crude product was obtained and purified by flash chromatography (silica gel) to give the pure carboxylic acid. **General experimental details**: NMR experiments were performed on a Bruker Avance III NMR spectrometer operating at 400 MHz (^1H) or 100 MHz (^{13}C). The deuterated solvent used was $CDCl_3$ unless otherwise specified. Chemical shifts were recorded in ppm. Spectra were calibrated by assignment of the residual solvent peak to δH 7.26 and δC 77.16 for $CDCl_3$. Coupling constants (*J*) were recorded in Hz. Infrared (IR) spectrometry was performed on a Shimadzu FTIR 8400 s spectrometer, with samples analyzed either as thin films on NaCl plates or using an ATR attachment. ESIMS analyses were conducted on a Thermo Scientific LTQ-Orbitrap mass spectrometer. EIMS analyses were performed using a Kratos Analytical Concept ISQ hybrid magnetic sector quadrupole tandem or Shimadzu GCMS-QP2010 mass spectrometers. Cyclic voltammetry studies were carried out using a Metrohm 797 VA potentiostiat fitted with a glassy carbon working electrode, a platinum auxiliary electrode and a saturated calomel reference electrode (SCE). Measurements were performed at RT in 20 mL of a 0.1 M tetrabutylammonium perchlorate solution in acetonitrile containing the naphthoquinones at 1 mM. The electrochemical cell was deoxygenated by purging with N2 for 2 min before scanning between –0.850 and 0.500 V (vs SCE) at 100 mV/s scan rate. The electrodes and measurement cell were rinsed with acetonitrile between each experiment. TLC was performed using Merck silica gel 60-F254 plates. Developed TLC plates were visualized by UV absorbance (254 nm) or through application of heat to a plate stained with cerium molybdate {$Ce(NH_4)_2(NO_3)_6$, $(NH_4)_6Mo_7O_{24}\cdot 4H_2O$, H_2SO_4, H2O}. Flash column chromatography was performed with flash grade silica gel (60 μm) and the indicated eluent in accordance with standard techniques. Melting points were obtained with a Stuart Scientific melting point SMP1 apparatus and are uncorrected. Optical rotations were obtained using a Rudolph research analytical Autopol III automatic polarimeter. UV-Vis absorption spectra were recorded in a 1.0 cm path length cuvette on a UV-1800 Shimadzu UV spectrophotometer. All fluorescence experiments were performed with a PerkinElmer LS55 spectrofluorometer in a 1.0 cm x 0.2 cm quartz cuvette. Unless otherwise specified, reactions were conducted with magnetic stirring under N_2, and all chemicals and reagents were purchased from Sigma-Aldrich, AK Scientific, Combi-blocks and Oakwood and used without purification. **Reference**: Betts, R.L.; Murphy, S.T.; Johnson, C.R. Enzymatic desymmetrization/resolution of epoxydiols derived from 1,4-naphthoquinone, 5-hydroxy-1,4-naphthoquinone and 5,8-dihydroxy-1,4-naphthoquinone. Tetrahedron: Asymmetry 2004, 15, 2853-2860, doi:https://doi.org/10.1016/j.tetasy.2004.07.051.

Table S2. Bioactivity profiles and physical properties of SCQs.

SCQ	ID	Cytoprotection				Effects on Metabolism-Related Markers												Redox Activity			
		Viability (%)				ATP (%)				Lactate (%)				BHB (%)				R-Total (ΔAb)		R-NQO1 (%)	R-Other (%)
		Mean	SD	*p*-Value vs RT		Mean	SD	*p*-Value vs RT		Mean	SD	*p*-Value vs NT		Mean	SD	*p*-Value vs NT		Mean	SD	Mean	Mean
1	UTAS#121	16.0	3.9	>0.99	ns	27.1	0.9	>0.99	ns	–	–	–	–	60.2	16.5	<0.001	***	–	–	–	–
2	UTAS#122	13.1	4.2	0.980	ns	29.6	7.0	>0.99	ns	–	–	–	–	191.4	0.5	<0.001	***	–	–	–	–
3	UTAS#125	16.9	4.6	>0.99	ns	22.0	8.4	>0.99	ns	–	–	–	–	85.3	15.4	0.94	ns	–	–	–	–
4	UTAS#120	20.1	4.0	>0.99	ns	27.9	2.9	>0.99	ns	–	–	–	–	78.7	24.3	0.35	ns	–	–	–	–
5	UTAS#124	22.0	4.6	>0.99	ns	29.4	6.7	>0.99	ns	–	–	–	–	77.9	18.9	0.29	ns	–	–	–	–
6	UTAS#123	82.8	9.5	<0.001	***	65.5	7.7	<0.001	***	–	–	–	–	121.5	14.5	0.34	ns	–	–	–	–
7	UTAS#128	14.3	4.1	>0.99	ns	21.6	2.6	0.980	ns	–	–	–	–	92.1	8.0	>0.99	ns	–	–	–	–
8	UTAS#127	17.7	5.7	>0.99	ns	17.5	0.9	0.600	ns	–	–	–	–	66.4	23.7	0.006	**	–	–	–	–
9	UTAS#129	18.6	6.8	>0.99	ns	19.3	4.7	0.870	ns	–	–	–	–	79.9	6.4	0.45	ns	–	–	–	–
10	UTAS#126	30.1	7.0	>0.99	ns	21.2	2.9	0.980	ns	–	–	–	–	93.6	4.5	>0.99	ns	–	–	–	–
11	UTAS#131	21.2	5.8	>0.99	ns	27.7	11.1	>0.99	ns	–	–	–	–	152.7	17.1	<0.001	***	–	–	–	–
12	UTAS#130	64.8	6.3	<0.001	***	64.8	9.2	<0.001	***	–	–	–	–	130.5	15.1	0.02	*	–	–	–	–
13	UTAS#39	65.9	8.3	<0.001	***	79.3	8.4	<0.001	***	128.7	7.0	0.04	*	117.2	15.7	0.74	ns	0.210	0.03	37.3	62.7
14	UTAS#113	73.4	5.3	<0.001	***	65.6	8.8	<0.001	***	–	–	–	–	146.2	5.9	<0.001	***	–	–	–	–
15	UTAS#114	33.9	7.1	>0.99	ns	54.2	8.9	0.090	ns	–	–	–	–	83.5	8.8	0.8	ns	–	–	–	–
16	UTAS#115	80.8	6.1	<0.001	***	67.2	3.5	<0.001	***	–	–	–	–	161.3	19.8	<0.001	***	–	–	–	–
17	UTAS#59	44.6	15.0	0.070	ns	79.9	20.2	<0.001	***	89.1	2.8	>0.99	ns	97.2	5.4	>0.99	ns	0.118	0.00	1.6	98.4
18	UTAS#50	47.0	10.0	0.040	*	27.3	6.9	>0.99	ns	114.8	2.6	0.93	ns	147.7	14.5	<0.001	***	0.103	0.00	0.5	99.5
19	UTAS#117	80.3	11.4	<0.001	***	40.4	3.1	>0.99	ns	–	–	–	–	76.3	5.1	0.2	ns	–	–	–	–
20	UTAS#64	69.4	14.5	<0.001	***	84.4	16.9	<0.001	***	82.6	5.3	0.72	ns	102.1	15.9	>0.99	ns	0.250	0.02	54.2	45.8
21	UTAS#51	55.3	13.8	<0.001	***	46.5	8.0	0.970	ns	72.1	22.4	0.06	ns	90.1	16.1	>0.99	ns	0.106	0.00	4.1	95.9
22	UTAS#6	42.9	5.5	0.600	ns	14.5	9.6	0.200	ns	113.1	3.9	0.98	ns	–	–	–	–	0.144	0.00	27.5	72.5
23	UTAS#21	33.9	5.6	>0.99	ns	19.6	6.1	0.900	ns	75.4	1.0	0.15	ns	148.1	20.7	<0.001	***	0.126	0.01	22.3	77.7
24	UTAS#5	51.0	5.8	0.010	*	6.6	1.5	0.002	**	132.8	11.2	0.009	**	165.9	19.9	<0.001	***	0.116	0.01	11.8	88.2
25	UTAS#1	5.6	1.0	<0.001	***	2.0	0.5	<0.001	***	99.6	6.3	>0.99	ns	86.3	19.6	0.98	ns	0.098	0.00	0.2	99.8
26	UTAS#19	24.9	1.5	>0.99	ns	4.2	0.9	<0.001	***	65.1	2.0	0.004	**	94.5	14.8	>0.99	ns	0.127	0.01	17.5	82.5
27	UTAS#26	56.8	6.0	<0.001	***	11.7	0.7	0.050	*	141.3	2.2	<0.001	***	123.8	24.5	0.19	ns	0.113	0.01	13.7	86.3
28	UTAS#15	52.2	10.4	0.004	**	22.4	4.6	>0.99	ns	99.2	5.6	>0.99	ns	79.6	15.3	0.43	ns	0.260	0.04	47.8	52.2
29	UTAS#4	34.1	2.2	>0.99	ns	3.4	0.1	<0.001	***	165.7	3.9	<0.001	***	90.9	3.1	>0.99	ns	0.119	0.01	10.7	89.3
30	UTAS#14	6.2	0.6	<0.001	***	2.1	0.4	<0.001	***	114.4	1.9	0.95	ns	108.4	11.7	>0.99	ns	0.104	0.00	1.4	98.6
31	UTAS#22	25.7	3.4	>0.99	ns	33.0	11.2	>0.99	ns	72.8	2.1	0.07	ns	89.3	12.5	>0.99	ns	0.154	0.01	30.6	69.4
32	UTAS#17	18.3	1.2	>0.99	ns	16.6	1.9	0.460	ns	123.9	6.4	0.19	ns	137.8	21.9	<0.001	***	0.150	0.01	32.6	67.4
33	UTAS#79	11.8	1.1	0.940	ns	78.2	7.3	<0.001	***	236.6	28.2	<0.001	***	1,132.1	4.3	<0.001	***	0.147	0.01	30.0	70.0
34	UTAS#41	51.7	3.7	0.010	**	73.5	4.8	<0.001	***	102.5	7.1	>0.99	ns	66.0	10.3	0.005	**	0.265	0.02	59.8	40.2
35	UTAS#61	100.7	28.4	<0.001	***	78.6	5.1	<0.001	***	104.6	4.0	>0.99	ns	131.4	3.7	0.02	*	0.361	0.07	67.5	32.5
36	UTAS#93	59.2	9.4	<0.001	***	82.7	8.1	<0.001	***	105.0	8.0	>0.99	ns	104.9	21.4	>0.99	ns	–	–	–	–
37	UTAS#28	32.0	3.3	>0.99	ns	13.3	1.3	0.110	ns	112.4	2.7	0.98	ns	97.7	15.9	>0.99	ns	0.123	0.01	10.5	89.5

38	UTAS#23	21.3	3.0	>0.99	ns	19.1	1.7	0.840	ns	103.4	1.2	>0.99	ns	181.2	19.0	<0.001	***	0.107	0.01	5.6	94.4
39	UTAS#67	74.9	18.5	<0.001	***	97.1	19.0	<0.001	***	67.4	1.7	0.01	**	135.7	0.9	0.002	**	0.178	0.02	37.4	62.6
40	UTAS#70	66.8	6.0	<0.001	***	91.8	20.2	<0.001	***	73.2	4.0	0.08	ns	109.0	3.2	>0.99	ns	0.123	0.01	18.6	81.4
41	UTAS#46	80.5	11.5	<0.001	***	53.7	7.1	0.120	ns	85.7	5.4	0.96	ns	111.1	15.4	>0.99	ns	0.162	0.01	31.8	68.2
42	UTAS#43	92.7	7.6	<0.001	***	46.6	5.7	0.970	ns	101.2	6.9	>0.99	ns	85.9	1.9	0.96	ns	0.133	0.01	12.1	87.9
43	UTAS#60	34.8	6.2	>0.99	ns	50.7	5.8	0.440	ns	97.0	15.8	>0.99	ns	142.5	10.8	<0.001	***	–	–	–	–
44	UTAS#29	10.3	2.3	<0.001	***	27.4	7.3	>0.99	ns	114.4	16.9	0.95	ns	124.2	17.8	0.17	ns	0.147	0.00	21.1	78.9
45	UTAS#30	17.7	0.6	>0.99	ns	11.8	2.7	0.050	*	112.9	3.1	0.98	ns	80.2	18.1	0.48	ns	0.118	0.01	6.5	93.5
46	UTAS#31	16.1	1.5	0.010	*	9.0	2.9	0.009	**	108.7	2.7	>0.99	ns	104.3	19.4	>0.99	ns	0.110	0.00	4.5	95.5
47	UTAS#32	15.7	1.2	0.010	*	15.4	2.0	0.290	ns	105.9	1.2	>0.99	ns	68.1	19.3	0.01	*	0.140	0.01	25.0	75.0
48	UTAS#33	15.1	1.4	0.006	**	9.6	1.5	0.010	*	103.8	3.8	>0.99	ns	113.9	19.2	0.97	ns	0.115	0.00	8.2	91.8
49	UTAS#34	18.9	1.1	>0.99	ns	17.8	11.6	0.650	ns	82.7	4.2	0.73	ns	93.5	12.7	>0.99	ns	0.108	0.00	3.6	96.4
50	UTAS#42	64.7	6.5	<0.001	***	79.1	9.3	<0.001	***	94.0	2.1	>0.99	ns	105.1	19.7	>0.99	ns	0.336	0.04	63.1	36.9
51	UTAS#18	15.2	2.6	0.007	**	3.5	1.6	<0.001	***	224.5	14.5	<0.001	***	311.8	21.9	<0.001	***	0.105	0.00	3.5	96.5
52	UTAS#25	56.7	6.8	<0.001	***	34.2	6.2	>0.99	ns	100.0	8.6	>0.99	ns	85.2	2.7	0.93	ns	0.265	0.02	54.6	45.4
53	UTAS#2	68.9	12.4	<0.001	***	6.4	1.5	0.001	**	112.4	10.0	0.98	ns	100.0	18.7	>0.99	ns	0.168	0.01	33.1	66.9
54	UTAS#8	52.5	7.3	0.004	**	19.8	4.8	0.920	ns	100.1	6.3	>0.99	ns	96.1	19.0	>0.99	ns	0.114	0.07	9.9	90.1
55	UTAS#16	48.2	5.9	0.060	ns	54.5	14.5	0.080	ns	121.2	2.3	0.36	ns	142.5	26.8	<0.001	***	0.505	0.08	68.5	31.5
56	UTAS#20	39.6	3.8	0.980	ns	44.5	5.6	>0.99	ns	78.5	3.4	0.34	ns	140.0	24.3	<0.001	***	0.355	0.05	66.4	33.6
57	UTAS#24	72.8	12.7	<0.001	***	47.4	14.2	0.920	ns	82.8	4.6	0.74	ns	125.2	21.1	0.13	ns	0.242	0.02	54.2	45.8
58	UTAS#40	21.3	10.2	>0.99	ns	79.9	5.8	<0.001	***	131.0	13.2	0.02	*	86.5	17.7	0.98	ns	0.814	0.15	82.1	17.9
59	UTAS#91	82.0	7.1	<0.001	***	77.4	8.8	<0.001	***	115.1	6.0	0.91	ns	145.0	12.2	<0.001	***	–	–	–	–
60	UTAS#82	64.1	2.9	<0.001	***	84.3	6.5	<0.001	***	132.7	1.8	0.009	**	156.1	16.2	<0.001	***	0.541	0.04	68.2	31.8
61	UTAS#73	86.2	9.4	<0.001	***	96.1	8.3	<0.001	***	99.2	6.9	>0.99	ns	76.8	13.6	0.22	ns	0.225	0.03	54.6	45.4
62	UTAS#81	84.2	19.9	<0.001	***	89.9	11.7	<0.001	***	140.4	20.4	<0.001	***	122.9	19.6	0.24	ns	0.350	0.07	68.9	31.1
63	UTAS#80	85.4	19.7	<0.001	***	89.1	10.9	<0.001	***	108.3	15.3	>0.99	ns	101.2	20.5	>0.99	ns	0.319	0.04	67.0	33.0
64	UTAS#62	95.5	13.7	<0.001	***	91.2	7.2	<0.001	***	90.2	2.9	>0.99	ns	164.3	10.9	<0.001	***	0.323	0.03	65.5	34.5
65	UTAS#78	80.5	21.1	<0.001	***	84.2	9.2	<0.001	***	85.1	1.7	0.93	ns	118.5	22.2	0.61	ns	0.245	0.04	55.0	45.0
66	UTAS#83	60.3	7.6	<0.001	***	73.5	16.0	<0.001	***	173.6	7.5	<0.001	***	257.5	14.5	<0.001	***	0.531	0.05	73.8	26.2
67	UTAS#84	74.4	3.8	<0.001	***	90.4	6.1	<0.001	***	103.5	7.2	>0.99	ns	129.8	20.3	0.03	*	0.440	0.02	71.2	28.8
68	UTAS#74	91.7	15.6	<0.001	***	100.7	16.0	<0.001	***	97.2	3.3	>0.99	ns	107.3	23.8	>0.99	ns	0.296	0.06	64.5	35.5
69	UTAS#88	91.8	9.8	<0.001	***	80.2	15.6	<0.001	***	111.1	11.2	>0.99	ns	177.1	20.6	<0.001	***	0.281	0.03	61.5	38.5
70	UTAS#89	85.2	9.5	<0.001	***	90.0	11.5	<0.001	***	97.5	3.4	>0.99	ns	106.0	8.5	>0.99	ns	0.252	0.04	57.0	43.0
71	UTAS#77	95.9	19.4	<0.001	***	99.1	19.2	<0.001	***	88.3	2.6	>0.99	ns	172.0	21.8	<0.001	***	0.306	0.06	60.9	39.1
72	UTAS#87	63.6	14.7	<0.001	***	79.4	19.9	<0.001	***	129.7	3.6	0.03	*	185.4	12.5	<0.001	***	0.444	0.06	73.0	27.0
73	UTAS#90	68.0	8.0	<0.001	***	87.6	13.1	<0.001	***	116.9	8.2	0.77	ns	153.1	18.9	<0.001	***	–	–	–	–
74	UTAS#86	49.5	10.7	0.150	ns	78.4	11.0	<0.001	***	124.7	7.9	0.15	ns	93.8	6.6	>0.99	ns	0.138	0.01	22.6	77.4
75	UTAS#85	61.4	9.6	<0.001	***	79.3	3.2	<0.001	***	118.4	5.2	0.62	ns	161.3	15.3	<0.001	***	0.276	0.02	46.6	53.4
76	UTAS#92	34.5	9.0	>0.99	ns	72.4	10.4	<0.001	***	159.2	5.0	<0.001	***	140.9	18.3	<0.001	***	–	–	–	–
77	UTAS#47	74.4	5.6	<0.001	***	76.4	5.2	<0.001	***	92.6	8.1	>0.99	ns	120.2	8.8	0.44	ns	0.478	0.11	56.2	43.8
78	UTAS#44	60.4	7.4	<0.001	***	80.5	14.0	<0.001	***	94.4	2.6	>0.99	ns	113.4	17.4	0.98	ns	0.356	0.05	66.0	34.0
79	UTAS#53	57.2	12.1	0.003	**	87.6	5.0	<0.001	***	114.4	14.1	0.95	ns	103.3	15.6	>0.99	ns	0.303	0.05	57.6	42.4
80	UTAS#95	87.6	9.4	<0.001	***	83.7	6.8	<0.001	***	–	–	–	–	130.8	13.9	0.02	*	–	–	–	–

81	UTAS#97	88.7	10.1	<0.001	***	81.1	8.4	<0.001	***	–	–	–	–	162.0	18.6	<0.001	***	–	–	–	–
82	UTAS#35	76.2	17.3	<0.001	***	76.7	7.6	<0.001	***	91.1	8.1	>0.99	ns	88.9	8.8	>0.99	ns	0.153	0.01	34.6	65.4
83	UTAS#65	60.1	13.5	<0.001	***	91.1	17.5	<0.001	***	103.0	2.6	>0.99	ns	104.6	2.8	>0.99	ns	0.250	0.03	56.4	43.6
84	UTAS#36	54.5	12.1	0.002	**	81.0	9.4	<0.001	***	90.1	5.0	>0.99	ns	184.0	22.4	<0.001	***	0.294	0.03	58.8	41.2
85	UTAS#71	70.5	19.5	<0.001	***	92.2	10.3	<0.001	***	112.3	23.7	0.98	ns	305.5	14.3	<0.001	***	0.173	0.01	40.7	59.3
86	UTAS#75	61.4	7.3	<0.001	***	95.4	18.6	<0.001	***	102.2	3.2	>0.99	ns	78.5	4.4	0.34	ns	0.166	0.02	37.1	62.9
87	UTAS#55	78.6	14.1	<0.001	***	94.8	4.1	<0.001	***	91.6	4.6	>0.99	ns	97.4	24.2	>0.99	ns	0.221	0.02	44.7	55.3
88	UTAS#57	60.0	26.9	<0.001	***	79.2	9.4	<0.001	***	99.5	2.6	>0.99	ns	115.2	18.9	0.91	ns	0.139	0.01	12.2	87.8
89	UTAS#49	23.8	4.8	>0.99	ns	89.3	16.4	<0.001	***	183.5	25.4	<0.001	***	82.9	9.3	0.75	ns	0.223	0.03	37.4	62.6
90	UTAS#48	53.6	22.5	0.020	*	56.6	13.5	0.020	*	111.3	8.1	>0.99	ns	97.9	27.3	>0.99	ns	0.157	0.01	24.1	75.9
91	UTAS#45	61.0	19.1	<0.001	***	45.9	8.3	0.980	ns	88.9	2.5	>0.99	ns	116.4	11.7	0.81	ns	0.117	0.01	1.9	98.1
92	UTAS#54	98.7	10.9	<0.001	***	66.1	12.6	<0.001	***	78.6	3.7	0.34	ns	101.0	5.2	>0.99	ns	0.119	0.01	7.3	92.7
93	UTAS#96	41.2	13.8	0.070	ns	56.6	9.2	0.020	*	–	–	–	–	133.8	16.4	0.006	**	–	–	–	–
94	UTAS#98	54.5	11.0	0.010	*	–	–	–	–	–	–	–	–	114.8	6.7	0.93	ns	–	–	–	–
95	UTAS#56	29.7	12.2	>0.99	ns	54.5	8.4	0.080	ns	82.7	9.8	0.73	ns	105.7	3.4	>0.99	ns	0.112	0.00	0.9	99.1
96	UTAS#66	66.9	3.9	<0.001	***	73.3	23.7	<0.001	***	65.8	2.1	0.005	**	117.7	3.3	0.69	ns	0.153	0.01	23.1	76.9
97	UTAS#37	100.3	17.3	<0.001	***	65.9	4.0	<0.001	***	80.6	7.7	0.52	ns	99.3	17.1	>0.99	ns	0.139	0.01	13.4	86.6
98	UTAS#94	65.2	6.3	<0.001	***	65.3	3.8	<0.001	***	92.9	5.5	>0.99	ns	107.5	13.9	>0.99	ns	–	–	–	–
99	UTAS#72	90.7	15.6	<0.001	***	84.8	29.5	<0.001	***	89.2	2.3	>0.99	ns	102.0	14.5	>0.99	ns	0.159	0.03	28.4	71.6
100	UTAS#76	68.1	14.1	<0.001	***	51.4	6.9	0.340	ns	97.4	5.2	>0.99	ns	160.8	21.2	<0.001	***	–	–	–	–
101	UTAS#38	63.9	3.5	<0.001	***	32.7	6.1	>0.99	ns	120.2	9.7	0.44	ns	–	–	–	–	0.104	0.00	1.0	99.0
102	UTAS#58	32.3	10.8	>0.99	ns	31.3	8.0	>0.99	ns	104.3	2.4	>0.99	ns	101.6	15.8	>0.99	ns	0.114	0.00	12.3	87.7
103	UTAS#69	3.3	0.3	0.100	ns	80.1	13.3	<0.001	***	71.1	4.4	0.04	*	188.2	8.4	<0.001	***	0.107	0.00	0.6	99.4

Table S2. *Cont.*

SCQ	Expression of Cytoprotective Proteins												Effects on Oxidative Damage												LogP	LogD
	Lin28A (%)				Hsp70 (%)				Acetyl-Tubulin (%)				BLP (%)				Nitrotyrosine (%)				γ-H2AX (%)					
	Mean	SD	*p*-Value vs NT		Mean	SD	*p*-Value vs NT		Mean	SD	*p*-Value vs NT		Mean	SD	*p*-Value vs NT		Mean	SD	*p*-Value vs NT		Mean	SD	*p*-Value vs NT			
1	101.0	1.4	>.999	ns	104.3	14.2	>.999	ns	97.3	3.7	>.999	ns	95.2	3.2	>0.99	ns	72.8	7.8	0.041	*	0.2	0.3	>0.99	ns	3.75	3.74
2	98.9	1.7	>.999	ns	99.0	4.8	>.999	ns	96.9	4.0	>.999	ns	93.6	1.9	>0.99	ns	78.0	4.6	0.110	ns	2.0	1.6	>0.99	ns	3.73	3.73
3	98.8	2.2	>.999	ns	93.1	1.5	0.998	ns	91.8	5.0	0.998	ns	97.4	4.5	>0.99	ns	86.0	17.9	0.930	ns	1.9	1.2	>0.99	ns	3.42	3.42
4	102.4	2.9	0.998	ns	97.3	3.3	>.999	ns	103.2	8.9	>.999	ns	95.0	3.1	>0.99	ns	79.7	8.2	0.216	ns	0.6	0.5	>0.99	ns	2.62	-0.24
5	100.4	2.4	>.999	ns	100.2	2.0	>.999	ns	84.8	0.8	0.145	ns	94.3	3.6	>0.99	ns	87.1	6.6	0.976	ns	0.7	0.6	>0.99	ns	3.74	0.49
6	98.2	1.0	0.999	ns	104.4	1.1	>.999	ns	96.9	8.1	>.999	ns	95.4	2.2	>0.99	ns	105.3	15.4	>.999	ns	0.9	0.4	>0.99	ns	4.94	4.94
7	101.0	6.2	>.999	ns	107.1	7.9	0.998	ns	101.9	4.0	>.999	ns	103.1	3.3	>0.99	ns	96.8	23.3	>.999	ns	0.7	0.4	>0.99	ns	2.14	2.14
8	99.2	3.0	>.999	ns	97.9	5.2	>.999	ns	94.7	9.4	0.999	ns	98.1	3.9	>0.99	ns	86.6	13.6	0.962	ns	0.9	0.7	>0.99	ns	2.13	2.13
9	104.6	3.7	0.376	ns	98.0	4.9	>.999	ns	113.8	8.0	0.389	ns	102.4	2.0	>0.99	ns	94.3	19.0	0.999	ns	1.5	0.3	>0.99	ns	1.82	1.82
10	97.6	3.7	0.998	ns	92.4	3.5	0.998	ns	94.7	7.3	0.999	ns	96.7	3.2	>0.99	ns	84.2	7.3	0.748	ns	0.5	0.4	>0.99	ns	1.02	-2.14
11	96.1	4.6	0.721	ns	92.0	2.6	0.992	ns	91.2	6.5	0.991	ns	100.0	6.6	>0.99	ns	73.6	10.0	<.001	***	1.7	1.3	>0.99	ns	2.14	-1.13
12	101.5	3.3	>.999	ns	101.3	5.1	>.999	ns	105.8	5.3	0.999	ns	99.9	2.3	>0.99	ns	75.5	3.5	0.122	ns	0.7	0.6	>0.99	ns	3.34	3.34
13	100.6	2.2	>.999	ns	126.5	7.1	<.001	***	116.7	3.6	0.070	ns	99.7	1.9	>0.99	ns	79.4	5.1	0.012	*	2.9	2.5	>0.99	ns	3.51	3.51
14	101.7	2.2	0.999	ns	99.5	3.2	>.999	ns	110.2	4.1	0.973	ns	103.4	1.4	>0.99	ns	82.5	9.0	0.520	ns	1.0	1.2	>0.99	ns	3.03	3.03
15	100.8	1.1	>.999	ns	104.3	12.7	>.999	ns	108.7	3.5	0.992	ns	106.4	6.2	>0.99	ns	82.9	10.4	0.573	ns	1.5	1.8	>0.99	ns	3.02	3.02
16	100.9	8.1	>.999	ns	106.0	6.7	0.983	ns	105.2	0.9	>.999	ns	105.8	8.3	>0.99	ns	90.7	17.3	0.979	ns	0.8	0.4	>0.99	ns	2.7	2.7
17	101.7	2.5	0.999	ns	149.9	11.9	<.001	***	106.4	5.7	0.999	ns	102.8	3.4	>0.99	ns	92.2	8.3	0.998	ns	4.4	1.1	0.520	ns	1.9	-1.37
18	101.8	1.5	0.999	ns	113.6	2.5	0.332	ns	106.9	3.4	0.999	ns	103.9	2.5	>0.99	ns	75.8	4.8	<.001	***	2.2	2.3	>0.99	ns	0.77	-2.53
19	97.8	4.0	0.998	ns	109.4	5.4	0.978	ns	129.6	9.0	<.001	***	104.5	4.4	>0.99	ns	89.9	14.1	0.998	ns	1.0	0.9	>0.99	ns	3.02	-0.24
20	102.1	1.6	0.998	ns	159.6	6.3	<.001	***	121.6	7.6	<.001	***	108.6	2.7	>0.99	ns	85.0	9.4	0.965	ns	3.7	2.0	0.930	ns	4.22	4.22
21	101.2	1.8	>.999	ns	119.6	11.8	0.005	**	104.7	3.4	>.999	ns	102.5	3.6	>0.99	ns	91.0	9.8	0.990	ns	2.1	1.3	>0.99	ns	2.04	2.04
22	–	–	–	–	–	–	–	–	–	–	–	–	–	–	–	–	–	–	–	–	–	–	–	–	4.51	4.51
23	99.2	2.4	>.999	ns	101.6	2.3	>.999	ns	133.1	4.1	<.001	***	99.9	6.9	>0.99	ns	92.0	7.2	0.999	ns	1.2	0.7	>0.99	ns	4.95	4.95
24	100.1	4.1	>.999	ns	108.4	2.7	0.991	ns	132.5	4.8	<.001	***	101.2	9.5	>0.99	ns	86.0	6.4	0.445	ns	5.0	2.4	0.270	ns	5.4	5.4
25	98.6	0.8	>.999	ns	102.3	6.8	>.999	ns	120.9	2.1	0.002	**	104.5	2.6	>0.99	ns	83.8	10.0	0.171	ns	1.3	0.9	>0.99	ns	6.29	6.29
26	99.2	1.1	>.999	ns	105.6	3.9	0.999	ns	111.4	16.1	0.851	ns	92.9	9.7	>0.99	ns	72.1	8.4	<.001	***	3.5	3.4	0.960	ns	3.91	3.91
27	104.4	1.4	0.467	ns	163.0	17.2	<.001	***	133.2	4.9	<.001	***	92.1	7.7	>0.99	ns	88.4	3.7	0.990	ns	3.1	3.5	>0.99	ns	5.53	5.53
28	–	–	–	–	–	–	–	–	–	–	–	–	98.3	6.3	>0.99	ns	–	–	–	–	–	–	–	–	3.32	3.32
29	98.4	3.8	0.999	ns	111.2	8.3	0.798	ns	113.7	6.9	0.407	ns	104.1	1.5	>0.99	ns	87.5	12.9	0.795	ns	4.8	1.6	0.330	ns	5.98	5.98
30	101.8	2.9	0.999	ns	126.8	1.9	<.001	***	93.9	8.4	0.998	ns	100.8	6.0	>0.99	ns	86.6	8.1	0.546	ns	1.5	1.6	>0.99	ns	9.04	9.04
31	–	–	–	–	–	–	–	–	–	–	–	–	–	–	–	–	–	–	–	–	–	–	–	–	4.33	4.33
32	100.3	3.0	>.999	ns	108.2	1.3	0.992	ns	110.0	1.9	0.977	ns	101.1	9.5	>0.99	ns	96.0	10.9	>.999	ns	1.7	1.2	>0.99	ns	4.75	4.75
33	102.6	0.9	0.980	ns	109.3	9.4	0.719	ns	116.1	15.9	0.001	**	114.7	8.6	0.94	ns	89.5	0.8	0.998	ns	1.8	0.2	>0.99	ns	4.55	4.55
34	100.1	1.4	>.999	ns	110.9	3.6	0.848	ns	128.3	1.9	<.001	***	99.8	1.1	>0.99	ns	78.8	9.1	0.007	**	3.4	2.3	0.980	ns	3.36	3.36
35	102.4	3.7	0.998	ns	105.9	3.4	0.999	ns	102.7	1.4	>.999	ns	108.6	5.2	>0.99	ns	78.5	6.1	0.012	*	3.0	1.3	>0.99	ns	1.71	1.71
36	100.6	2.4	>.999	ns	101.8	5.6	>.999	ns	107.2	2.6	0.998	ns	98.8	2.9	>0.99	ns	74.7	9.7	<.001	***	1.0	1.1	>0.99	ns	1.03	1.03
37	99.8	1.4	>.999	ns	142.4	27.7	<.001	***	124.7	11.3	<.001	***	98.3	2.8	>0.99	ns	76.2	2.1	0.002	**	1.7	1.7	>0.99	ns	1.85	-1.4

38	100.9	1.7	>.999	ns	108.9	5.9	0.982	ns	114.0	5.2	0.354	ns	98.8	4.6	>0.99	ns	100.1	9.1	>.999	ns	2.2	1.4	>0.99	ns	2.3	-0.93
39	100.0	0.8	>.999	ns	117.9	18.0	0.019	*	116.6	4.7	0.075	ns	104.5	0.5	>0.99	ns	75.6	6.8	<.001	***	1.3	0.4	>0.99	ns	2.74	-0.49
40	103.2	1.9	0.971	ns	105.1	1.0	0.999	ns	132.3	8.8	<.001	***	113.2	6.2	0.98	ns	82.1	13.9	0.068	ns	0.9	1.1	>0.99	ns	3.19	0.02
41	101.2	0.8	>.999	ns	162.3	23.4	<.001	***	102.8	2.3	>.999	ns	99.6	4.5	>0.99	ns	107.9	16.5	0.998	ns	4.9	3.5	0.300	ns	1.88	-1.43
42	100.6	2.5	>.999	ns	106.3	2.8	0.999	ns	111.1	3.9	0.892	ns	103.6	3.8	>0.99	ns	82.6	12.5	0.090	ns	1.5	0.5	>0.99	ns	2.04	-1.32
43	99.3	2.7	>.999	ns	104.0	2.1	>.999	ns	106.5	4.0	0.999	ns	91.5	7.0	>0.99	ns	83.5	6.3	0.212	ns	6.0	1.9	0.050	*	3.15	-0.08
44	99.8	1.8	>.999	ns	118.1	5.4	0.017	*	121.3	12.0	0.002	**	94.3	6.3	>0.99	ns	78.0	5.0	0.004	**	3.5	2.9	0.980	ns	2.36	2.36
45	101.6	3.5	0.999	ns	106.7	1.5	0.998	ns	121.2	15.5	0.002	**	103.2	5.5	>0.99	ns	85.8	5.9	0.414	ns	2.9	2.7	>0.99	ns	3.32	3.32
46	98.6	2.2	>.999	ns	106.4	1.8	0.999	ns	117.6	13.7	0.037	*	100.1	5.2	>0.99	ns	82.3	8.3	0.493	ns	3.2	2.5	0.980	ns	4.21	4.21
47	98.2	3.2	0.999	ns	106.7	2.6	0.998	ns	123.1	16.1	<.001	***	93.0	7.8	>0.99	ns	69.8	8.4	<.001	***	2.2	1.9	>0.99	ns	2.8	2.8
48	101.3	2.6	>.999	ns	104.1	5.4	>.999	ns	121.2	15.0	0.002	**	–	–	–	–	91.6	5.9	0.999	ns	2.5	1.1	>0.99	ns	3.77	3.77
49	100.9	1.7	>.999	ns	101.0	3.9	>.999	ns	115.3	15.5	0.174	ns	106.3	4.0	>0.99	ns	106.5	12.4	0.999	ns	3.7	1.0	0.910	ns	4.66	4.66
50	100.5	0.9	>.999	ns	107.6	3.3	0.998	ns	105.5	2.9	>.999	ns	101.4	4.7	>0.99	ns	78.7	14.0	0.007	**	2.6	1.7	>0.99	ns	3.23	3.23
51	101.8	2.3	0.999	ns	121.1	3.5	0.001	**	126.3	16.5	<.001	***	95.0	4.3	>0.99	ns	96.7	16.6	>.999	ns	3.6	1.3	0.950	ns	6.04	6.04
52	102.2	1.5	0.998	ns	136.5	12.8	<.001	***	112.5	16.5	0.649	ns	98.8	8.5	>0.99	ns	81.4	5.7	0.044	*	4.5	2.5	0.520	ns	2.18	2.18
53	102.2	1.3	0.998	ns	103.8	6.8	>.999	ns	129.9	3.9	<.001	***	100.3	5.1	>0.99	ns	111.8	19.9	0.817	ns	2.0	1.6	>0.99	ns	4.85	4.85
54	102.2	1.7	0.998	ns	160.0	10.0	<.001	***	127.6	1.7	<.001	***	98.7	4.7	>0.99	ns	96.5	13.6	>.999	ns	7.3	3.4	0.002	**	4.94	4.94
55	98.2	1.1	0.999	ns	109.8	10.2	0.964	ns	101.0	9.5	>.999	ns	100.6	8.8	>0.99	ns	81.4	9.8	0.124	ns	4.1	3.0	0.710	ns	2.02	2.02
56	–	–	–	–	–	–	–	–	–	–	–	–	100.3	8.8	>0.99	ns	–	–	–	–	–	–	–	–	3.55	3.55
57	99.7	2.5	>.999	ns	110.2	5.4	0.367	ns	125.0	13.0	<.001	***	98.9	8.6	>0.99	ns	76.8	6.7	0.002	**	2.4	2.3	>0.99	ns	2.15	2.15
58	103.8	3.8	0.770	ns	167.7	16.7	<.001	***	114.3	4.3	0.304	ns	97.7	6.3	>0.99	ns	118.1	17.7	0.012	*	3.7	2.3	0.920	ns	2.59	1.51
59	104.5	2.0	0.420	ns	114.2	9.5	0.242	ns	108.0	8.7	0.999	ns	95.3	9.1	>0.99	ns	78.0	10.6	0.008	**	0.3	0.4	>0.99	ns	3.04	3.04
60	101.8	1.6	0.999	ns	110.2	6.2	0.934	ns	111.7	2.4	0.803	ns	86.5	6.9	0.98	ns	86.3	15.5	0.495	ns	0.6	0.4	>0.99	ns	3.73	3.73
61	101.1	0.4	>.999	ns	111.4	4.9	0.103	ns	117.6	2.4	0.037	*	104.3	2.1	>0.99	ns	91.5	19.9	0.991	ns	0.3	0.5	>0.99	ns	3.83	3.83
62	103.8	0.6	0.770	ns	107.9	5.1	0.998	ns	106.7	8.2	0.999	ns	95.0	4.6	>0.99	ns	85.1	10.6	0.313	ns	0.4	0.6	>0.99	ns	2.81	2.81
63	102.3	1.9	0.998	ns	106.0	3.5	0.999	ns	108.9	2.5	0.991	ns	110.5	9.9	>0.99	ns	71.2	8.8	<.001	***	0.8	0.6	>0.99	ns	2.81	2.81
64	99.8	1.2	>.999	ns	109.1	4.5	0.981	ns	103.6	3.4	>.999	ns	94.8	3.3	>0.99	ns	78.9	11.6	0.008	**	2.7	1.4	>0.99	ns	3.1	3.1
65	102.5	2.0	0.998	ns	98.7	1.6	>.999	ns	108.9	3.1	0.991	ns	112.4	7.6	0.98	ns	95.9	20.3	>.999	ns	0.8	0.8	>0.99	ns	3.1	3.1
66	104.0	1.6	0.670	ns	111.9	12.4	0.028	*	112.7	7.7	0.607	ns	105.7	5.0	>0.99	ns	91.1	3.9	0.998	ns	0.2	0.3	>0.99	ns	3.54	3.53
67	101.9	1.0	0.999	ns	112.2	8.3	0.601	ns	109.6	14.8	0.340	ns	94.7	10.0	>0.99	ns	103.6	21.0	>.999	ns	0.7	0.2	>0.99	ns	3.99	3.99
68	103.2	1.0	0.971	ns	105.9	5.5	0.999	ns	103.3	4.8	>.999	ns	93.9	3.3	>0.99	ns	84.4	7.5	0.229	ns	1.1	0.6	>0.99	ns	3.43	3.43
69	103.1	4.6	0.977	ns	98.9	5.9	>.999	ns	112.8	4.7	0.587	ns	91.8	8.4	>0.99	ns	97.3	9.3	>.999	ns	0.4	0.5	>0.99	ns	3.87	3.87
70	100.6	1.8	>.999	ns	108.3	10.2	0.608	ns	117.0	13.6	0.001	**	96.9	3.1	>0.99	ns	99.8	14.3	>.999	ns	0.7	0.9	>0.99	ns	4.31	4.31
71	103.5	0.8	0.895	ns	103.6	7.2	>.999	ns	110.3	5.3	0.965	ns	91.7	1.5	>0.99	ns	87.2	13.6	0.652	ns	3.1	2.9	>0.99	ns	3.41	3.41
72	100.8	2.3	>.999	ns	104.2	1.8	>.999	ns	101.5	11.2	>.999	ns	98.8	4.1	>0.99	ns	84.9	10.3	0.379	ns	0.6	0.7	>0.99	ns	3.86	3.86
73	101.5	2.2	>.999	ns	108.8	6.7	0.983	ns	104.0	10.8	>.999	ns	94.6	8.9	>0.99	ns	82.3	8.9	0.076	ns	0.9	0.6	>0.99	ns	4.3	4.3
74	107.0	3.9	0.006	**	121.7	9.9	<.001	***	86.2	7.8	0.038	*	99.8	3.8	>0.99	ns	107.5	18.8	0.998	ns	1.2	0.3	>0.99	ns	0.86	0.86
75	103.1	4.3	0.977	ns	101.4	6.0	>.999	ns	99.2	12.2	>.999	ns	92.1	3.3	>0.99	ns	82.4	15.5	0.081	ns	1.0	1.4	>0.99	ns	1.28	1.28
76	102.0	1.6	0.998	ns	116.7	4.9	0.048	*	99.6	7.0	>.999	ns	106.9	5.6	>0.99	ns	93.7	17.7	0.982	ns	0.6	0.3	>0.99	ns	0.92	0.92
77	102.1	1.5	0.998	ns	114.1	7.2	0.256	ns	108.8	2.8	0.991	ns	95.3	4.9	>0.99	ns	82.8	12.2	0.101	ns	2.8	2.2	>0.99	ns	2.39	2.39
78	101.0	5.4	>.999	ns	106.8	3.6	0.998	ns	109.6	2.0	0.981	ns	101.3	5.5	>0.99	ns	96.4	19.9	>.999	ns	1.3	0.8	>0.99	ns	3.93	3.39
79	101.3	2.3	>.999	ns	125.8	22.3	<.001	***	119.3	4.8	0.010	**	101.1	1.5	>0.99	ns	76.5	6.5	0.056	ns	2.4	2.3	>0.99	ns	4.22	4.22
80	100.2	3.2	>.999	ns	97.5	3.1	>.999	ns	100.9	5.6	>.999	ns	107.0	4.7	>0.99	ns	99.9	17.2	>.999	ns	1.1	0.8	>0.99	ns	3.28	3.28

81	106.0	3.2	0.047	*	105.3	3.0	0.999	ns	96.6	9.3	>.999	ns	94.7	6.5	>0.99	ns	96.3	24.5	>.999	ns	1.7	0.9	>0.99	ns	3.28	3.28
82	100.5	0.6	>.999	ns	106.4	4.0	0.999	ns	106.5	7.3	0.998	ns	105.4	0.7	>0.99	ns	95.0	17.8	>.999	ns	2.9	4.4	>0.99	ns	3.56	3.56
83	100.2	1.9	>.999	ns	142.3	13.8	<.001	***	134.5	7.4	<.001	***	103.9	8.0	>0.99	ns	79.5	5.0	0.201	ns	2.4	2.7	>0.99	ns	4.17	4.17
84	105.0	3.2	0.229	ns	126.6	2.9	<.001	***	117.4	2.2	0.043	*	91.0	8.9	>0.99	ns	105.1	20.3	0.999	ns	2.7	0.9	>0.99	ns	4.62	4.62
85	103.4	1.2	0.925	ns	110.2	4.9	0.934	ns	117.5	1.9	0.040	*	100.8	2.1	>0.99	ns	92.3	19.2	0.998	ns	2.2	2.2	>0.99	ns	5.06	5.06
86	104.4	1.6	0.672	ns	105.6	2.3	0.999	ns	113.4	13.2	0.464	ns	111.5	6.2	>0.99	ns	81.2	11.0	0.200	ns	2.4	1.4	>0.99	ns	5.51	5.51
87	100.1	1.5	>.999	ns	104.3	3.0	>.999	ns	104.3	3.6	>.999	ns	98.1	4.7	>0.99	ns	83.8	5.1	0.171	ns	1.9	1.4	>0.99	ns	4.31	4.31
88	–	–	–	–	–	–	–	–	–	–	–	–	–	–	–	–	–	–	–	–	–	–	–	–	6.75	6.75
89	–	–	–	–	–	–	–	–	–	–	–	–	–	–	–	–	–	–	–	–	–	–	–	–	5.27	5.27
90	102.1	1.0	0.998	ns	106.9	3.7	0.998	ns	102.0	6.4	>.999	ns	97.0	5.8	>0.99	ns	71.5	5.2	<.001	***	0.9	0.9	>0.99	ns	1.19	-2.18
91	99.8	3.7	>.999	ns	104.1	1.8	>.999	ns	105.6	1.4	0.999	ns	99.6	5.9	>0.99	ns	86.7	10.5	0.668	ns	1.4	1.5	>0.99	ns	2.73	-0.58
92	98.5	3.9	>.999	ns	104.4	3.8	>.999	ns	111.4	3.9	0.851	ns	100.8	2.9	>0.99	ns	77.4	8.2	0.003	**	2.9	1.4	>0.99	ns	3.02	-0.26
93	100.8	4.1	>.999	ns	104.0	2.4	>.999	ns	93.3	8.0	0.998	ns	102.4	8.0	>0.99	ns	76.4	9.7	0.001	**	0.5	1.0	>0.99	ns	3.13	-0.23
94	98.3	1.3	0.999	ns	92.9	3.9	0.998	ns	109.9	5.8	0.629	ns	100.8	6.7	>0.99	ns	79.6	15.5	0.025	*	1.2	1.1	>0.99	ns	3.13	-0.23
95	98.5	1.6	>.999	ns	102.4	4.7	>.999	ns	97.7	13.3	>.999	ns	100.5	1.9	>0.99	ns	77.2	4.7	0.005	**	1.9	0.8	>0.99	ns	3.12	-0.26
96	100.4	1.3	>.999	ns	110.1	8.2	0.943	ns	103.2	3.9	>.999	ns	106.8	3.7	>0.99	ns	69.3	5.0	<.001	***	1.6	1.4	>0.99	ns	2.97	-0.39
97	100.2	1.4	>.999	ns	111.4	6.6	0.762	ns	103.6	8.9	>.999	ns	91.6	4.0	>0.99	ns	77.3	7.0	0.005	**	2.9	1.2	>0.99	ns	3.42	0.12
98	100.5	2.3	>.999	ns	102.0	1.9	>.999	ns	94.1	10.0	0.999	ns	102.3	5.0	>0.99	ns	78.4	7.5	0.011	*	2.2	0.8	>0.99	ns	3.42	0.12
99	103.2	1.9	0.971	ns	105.6	2.8	0.999	ns	112.2	2.5	0.709	ns	102.5	7.4	>0.99	ns	86.9	10.9	0.701	ns	1.5	0.2	>0.99	ns	3.86	0.74
100	103.0	1.3	0.979	ns	107.5	6.6	0.998	ns	114.5	12.0	0.274	ns	104.2	0.8	>0.99	ns	75.0	11.0	<.001	***	3.2	0.7	0.980	ns	4.31	1.25
101	100.5	2.7	>.999	ns	98.4	7.0	>.999	ns	96.2	6.7	>.999	ns	111.0	8.4	>0.99	ns	73.8	8.6	<.001	***	1.7	1.1	>0.99	ns	5.53	5.53
102	101.3	5.4	>.999	ns	101.4	3.1	>.999	ns	105.8	3.4	0.999	ns	101.2	1.4	>0.99	ns	82.7	8.7	0.224	ns	1.7	1.8	>0.99	ns	2.31	-4.03
103	94.7	2.0	0.149	ns	130.9	18.2	<.001	***	92.1	6.6	0.998	ns	108.8	4.2	>0.99	ns	121.1	10.0	0.033	*	46.0	10.8	<0.001	***	0.87	0.87

Compound color represents different chemical classes: **aliphatic**, **amino alcohol**, **acid**, **amino acid**, **aliphatic ester**, **amino ester**, and **slight polarity**. Data represents the mean ± standard deviation (SD) of multiple independent experiments using the human hepatocarcinoma cell line HepG2 or the predicted partition coefficient (logP, ChemDraw Professional, version 16.0, PerkinElmer, Waltham, MA, USA) and distribution coefficient (logD, MarvinView, version 19.25, ChemAxon, Budapest, Hungary) values. For some compounds, not all parameters could be assessed. One- or two-way ANOVA followed by Dunnett's multiple comparison post-test was performed using GraphPad Prism (version 8.2.1, San Diego, CA, USA) to compare test compounds and control(s) or between chemical classes: *** $p < 0.001$, ** $p < 0.01$, * $p < 0.05$, otherwise non-significant (ns). RT, rotenone-treated; NT, non-treated; BHB, β-hydroxybutyrate; R-Total, total reduction of quinone; R-NQO1, reduction of quinone by NQO1; R-Other, reduction by other reductases; BLP, basal lipid peroxidation; γ-H_2AX, γ-H_2AX-positive cells.

Table S3. Bioactivity profiles and physical properties of SCQs belonging to different chemical classes.

Parameter		Aliphatic			Amino Alcohol			Acid			Amino Acid			Aliphatic Ester			Amino Ester			Slight Polarity		
		**Mean**	**SD**	**N**	**Mean**	**SD**	**N**	**Mean**	**SD**	**N**	**Mean**	**SD**	**N**	**Mean**	**SD**	**N**	**Mean**	**SD**	**N**	**Mean**	**SD**	**N**
Cytoprotection	Viability (%)	36.3	20.4	15	62.0	29.0	29	46.8	21.8	11	61.0	27.5	15	15.6	3.0	6	68.6	11.1	17	40.2	23.5	10
Metabolism-Related Markers	ATP (%)	26.7	27.9	15	70.0	26.5	29	48.0	30.9	11	53.6	15.8	14	15.2	6.9	6	80.0	12.3	17	46.0	30.7	10
	Lactate (%)	119.4	42.1	15	115.0	23.1	20	93.7	16.6	9	83.5	14.9	10	104.7	11.5	6	95.4	11.4	13	120.5	48.8	10
	BHB (%)	187.7	285.2	13	130.1	44.7	29	114.6	29.3	11	112.3	25.7	15	97.4	21.0	6	126.7	53.0	17	135.8	70.4	10
Redox Activity	R-Total (ΔAb)	0.15	0.05	15	0.33	0.11	16	0.13	0.03	8	0.14	0.02	8	0.12	0.02	6	0.25	0.10	13	0.29	0.22	10
	R-NQO1 (%)	22.9	17.4	15	61.1	12.7	16	14.8	13.6	8	13.9	10.4	8	11.5	9.2	6	45.1	19.1	13	41.0	28.9	10
	R-Other (%)	77.1	17.4	15	38.9	12.7	16	85.2	13.6	8	86.1	10.4	8	88.5	9.2	6	54.9	19.1	13	59.0	28.9	10
Cytoprotective Proteins	Lin28A (%)	100.5	1.7	12	102.1	2.5	29	100.9	1.5	11	99.9	1.9	15	100.1	1.4	6	101.7	2.0	16	100.6	2.9	8
	Hsp70 (%)	114.4	17.7	12	105.8	6.3	29	117.6	23.4	11	104.1	5.6	15	107.2	5.8	6	114.8	16.6	16	130.0	23.7	8
	Acetyl-Tubulin (%)	117.2	13.4	12	105.2	7.4	29	109.2	11.8	11	105.1	10.9	15	120.0	2.9	6	110.2	10.0	16	116.1	13.8	8
Oxidative Damage	BLP (%)	101.5	6.2	13	99.3	6.4	29	101.5	4.9	11	100.0	4.6	15	99.4	5.7	5	101.1	5.5	16	99.9	3.8	9
	Nitrotyrosine (%)	84.5	7.2	12	87.1	9.0	29	85.0	10.7	11	79.6	6.2	15	85.7	12.6	6	89.0	9.9	16	98.0	17.5	8
	γ-H_2AX (%)	2.67	1.33	12	1.06	0.79	29	2.06	1.43	11	1.93	1.39	15	2.99	0.60	6	2.12	0.80	16	9.20	14.96	8
LogP		5.00	1.45	15	2.92	1.02	29	2.18	0.82	11	3.00	0.77	15	3.52	0.87	6	4.05	1.15	17	3.45	1.73	10
LogD		5.00	1.45	15	2.92	1.02	29	-1.31	1.24	11	-0.29	0.83	15	3.52	0.87	6	4.02	1.16	17	3.34	1.82	10

Data represents mean ± SD of average responses from compounds belonging to the same chemical class using the human hepatocarcinoma cell line HepG2, N = number of compounds tested for the parameter. BHB, β-hydroxybutyrate; BLP, basal lipid peroxidation; R-Total, total reduction of quinone; R-NQO1, reduction of quinone by NQO1; R-Other, reduction by other reductases; γ-H2AX, γ-H_2AX-positive cells.
